# Design and heterologous expression of a novel dimeric LL37 variant in *Pichia pastoris*

**DOI:** 10.1186/s12934-021-01635-x

**Published:** 2021-07-23

**Authors:** Na Zhan, Licong Zhang, Hong Yang, Yalan Zheng, Xinke Wei, Jiajun Wang, Anshan Shan

**Affiliations:** Institute of Animal Nutrition, Northeast Agricultural University, No. 600 Changjiang Road, Xiangfang District, Harbin, China

**Keywords:** Antimicrobial peptide, LL37, *Pichia pastoris*, Dimeric, Fusion expression

## Abstract

**Background:**

The antimicrobial peptide LL37 is produced by white blood cells (mainly neutrophils) and various epithelial cells, and has the outstanding advantages of participating in immune regulation, causing chemotaxis of immune cells and promoting wound healing. However, the central domain of LL37 needs to be improved in terms of antimicrobial activity.

**Results:**

In this study, the amino acid substitution method was used to improve the antimicrobial activity of the LL37 active center, and a dimeric design with a better selection index was selected. A flexible linker was selected and combined with the 6 × His-SUMO tag and LG was successfully expressed using *Pichia pastoris* as a host. Recombinant LG displayed strong antimicrobial activity by destroying the cell membrane of bacteria but had low hemolytic activity. In addition, compared with monomeric peptide FR, rLG had improved ability to tolerate salt ions.

**Conclusion:**

This research provides new ideas for the production of modified AMPs in microbial systems and their application in industrial production.

**Supplementary Information:**

The online version contains supplementary material available at 10.1186/s12934-021-01635-x.

## Background

While the abuse and excessive use of antibiotics in animal husbandry production leads to the emergence of drug-resistant strains, their residual problems also adversely affect the safety of dairy and meat products, and threaten human health [1–3]. With the gradual deepening of research, antimicrobial peptides (AMPs) with the characteristics of broad-spectrum antimicrobial, and unique mechanism of action can avoid the negative effects of traditional antibiotics in animal husbandry, and therefore have great potential to become a new feed additive [4–7]. As the only member of the human cathelicidin family, LL37 produced by white blood cells (mainly neutrophils) and various epithelial cells is widely found in various human tissues and body fluids [8–11]. Due to its outstanding advantages of participating in immune regulation, causing immune cell chemotaxis, and promoting wound healing, LL37 has maintained high research interest in recent years [12]. In addition, it displays defense against many types of pathogens, including bacteria, fungi, viruses, parasites and even cancer cells [13]. A series of studies showed that the helix structure region of residues 17–29 was the key domain for the biological functions of LL37 [14, 15]. To further improve the biological activity of this domain, several strategies, such as amino acid substitution and sequence hybridization, have been employed. Tan et al. hybridized this domain with FV7 (FRIRVRV-NH_2_), which effectively enhanced its antimicrobial activity [16].

At present, the method for obtaining modified AMPs mainly depends on the chemical synthesis method, and the high cost of synthesis greatly limits its application in animal husbandry production [17]. The use of microbial recombinant expression of modified AMPs seems to be a viable means to overcome this barrier. As a eukaryote, *Pichia pastoris*, methylotrophic yeast, has the advantages of posttranslational processing and modification and can secrete the produced heterologous protein into the culture medium, which also facilitates the separation and purification of recombinant protein in the later stage [18]. In addition, the target gene can be directly integrated with the yeast genome, which helps stabilize the expression of the target protein [19]. However, modified AMPs often have the characteristics of small molecular weight and easy degradation by microbial protease, which makes the expression of recombinant modified peptides in microbial systems more challenging [20]. The fusion or tandem repeated expression of AMPs provides a solution to this problem. The fusion linker peptide strategy may provide other advantages for the fusion protein, such as improving biological activity and expanding expression yield [21]. Commonly, linkers are grouped into two categories: flexible linkers and rigid linkers [22]. However, for the design of fusion proteins, the choice of linker peptide is not fixed. The results of Fan and coworkers showed that compared with other linkers, rigid linkers could significantly improve the natural structure and biological activity of the fusion protein VRT [23]. Zhang et al. found that when connecting two enzymes (FDH and LeuDH), F-R-L (with rigid linker) could fold independently and ensure structural stability, while F-S-L (with flexible linker) achieved the best cofactor channel effect due to the tightness of the domain [24]. Therefore, the rational design of linkers plays an important role to guarantee the biological activity of the protein.

In this study, the specific amino acid of the LL37 active center was replaced to improve the antimicrobial activity. To further improve the antimicrobial activity of this modified peptide and facilitate its recombinant expression in *P. pastoris*, this study evaluated the effects of three designs (direct connection, rigid linker connection and flexible linker connection) on the antimicrobial activity and hemolytic toxicity of the formed dimeric. It is hoped that this tandem expression strategy can provide new ideas for the production of modified AMPs in microbial systems and the application of modified AMPs in industrial production.

## Results and discussion

### Antimicrobial activity and hemolytic activity of peptides

The antimicrobial activity of the peptides was shown in Table 1. The antimicrobial activity of FR (FKRIVQRIKRFLR) obtained by replacing aspartic acid (D) with arginine (R) were significantly improved compared with FD (FKRIVQRIKDFLR). Wang et al. pointed out that the cations of AMPs are important parameters that affect the antimicrobial activity of AMPs [25]. The positive charges of AMPs can interact electrostatically with the negatively charged components on the surface of microbial cells, resulting in disturbance of cell membrane permeability and cell death [26]. The presence of lysine (K), R and histidine (H) is the reason why AMPs are positively charged. H is an amphiphilic dissociative amino acid that is easily affected by the environment. AMPs composed of H are often inferior to AMPs composed of K and R in terms of antimicrobial activity, so H is not commonly used in the study of AMPs modification, while K and R are common in the molecular design of AMPs. Although K and R have the same charge, AMPs containing R are usually better than AMPs containing K in terms of antimicrobial activity when the number of K and R in different AMPs keep the same. This may be due to their different membrane binding properties. The guanidine group in the side chain of R has the remarkable ability to establish strong hydrogen bonds with the double-layer phospholipids of the cell membrane, while the side chain of K can only interact with a single lipid head group [27]. And once it enters the cell, the affinity of R-containing peptides for DNA seems to be higher than that of K-containing peptides [28]. Therefore, in this study, in order to improve the antimicrobial activity of FD, the amino acid R was preferentially selected to replace the original amino acid D. In addition, previous studies have shown that within a certain range, the increase in the total cationic charge of AMPs will significantly enhance the antimicrobial ability of peptides [29]. Similar to the results of this study, Gong et al. replaced the uncharged alanine (A) in DRP-AC4b with K, and the number of charges increased from + 3 to + 4, thereby reducing the MIC value from 21.53 to 14.49 μM [30].

**Table 1:** The MIC (μM) values of peptides

	MIC				
	FD	FR	(FR)_2_	LG	LA
Gram-bacteria					
*E. coli* ATCC 25922	32.00 ± 0.00	7.11 ± 1.66	1.22 ± 0.41	2.22 ± 0.63	2.22 ± 0.63
*E. coli* ATCC 078	16.00 ± 0.00	8.00 ± 0.00	1.11 ± 0.31	1.22 ± 0.41	2.44 ± 0.83
*E. coli* UB 1005	16.00 ± 0.00	2.44 ± 0.83	1.00 ± 0.00	1.22 ± 0.41	1.11 ± 0.31
*S. Typhimurium* C 7731	32.00 ± 0.00	8.00 ± 0.00	1.11 ± 0.31	1.22 ± 0.41	1.11 ± 0.31
*S. Typhimurium* ATCC 14028	32.00 ± 0.00	8.00 ± 0.00	2.22 ± 0.63	4.00 ± 0.00	4.44 ± 1.25
*P. aeruginosa* ATCC 27853	> 32	16.00 ± 0.00	2.22 ± 0.63	4.00 ± 0.00	4.44 ± 1.25
Gram + bacteria					
*S. aureus* ATCC 29213	> 32	8.00 ± 0.00	2.00 ± 0.00	2.00 ± 0.00	> 32
*S. epidermidis* ATCC 12228	> 32	3.78 ± 0.63	2.00 ± 0.00	2.00 ± 0.00	> 32
*S. aureus* ATCC 25923	> 32	16.00 ± 0.00	3.78 ± 0.63	3.78 ± 0.63	> 32
*S. faecalis* ATCC 29212	> 32	4.00 ± 0.00	2.00 ± 0.00	2.22 ± 0.63	> 32

**Fig. 1:**
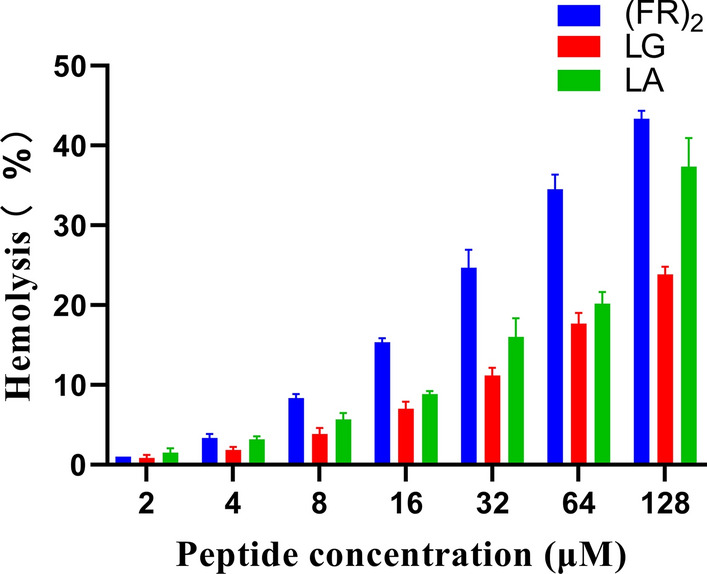
Hemolytic activity assay of peptides against human red blood cells

The modified AMP FR contained 13 amino acids and had the characteristics of a small molecular weight (1.76 kDa), which resulted in great difficulties with the separation, purification and identification of its recombinant expression. To overcome these obstacles, the method of fusing two domains together to form a new dimeric protein might be a good choice. Wang and coworkers connected PMAP-36 in an antiparallel manner to form a dimer (PMAP-36)_2_ and found that (PMAP-36)_2_ had strong resistance to Gram- and Gram + bacteria in vitro and did not show hemolytic activity [31]. However, in this study, the fusion protein (FR)_2_ obtained by direct connection had the highest antimicrobial activity and showed the strongest toxicity (Table 1 and Fig. 1). At the highest peptide concentration (128 μM), (FR)_2_ could destroy approximately 43% of blood cells, so its safety in product applications cannot be guaranteed (Fig. 1). Therefore, a suitable linker peptide is essential for the required function. Rigid linkers usually maintain a certain distance between protein domains and prevent adverse interactions between the domains [32]. In contrast, flexible linker peptides are rich in amino acids such as glycine (G) and serine (S), which usually increase the flexibility of the domain and improve the folding of the fusion protein [21, 33]. In this study, the LG (FKRIVQRIKRFLRGGGGSFKRIVQRIKRFLR) formed by the flexible linker peptide GGGGS was superior to the LA (FKRIVQRIKRFLRAEAAAKAFKRIVQRIKRFLR) formed by the rigid linker peptide AEAAAKA in terms of antimicrobial activity and toxicity (Table 1 and Fig. 1).

The three designs ((FR)_2_, LG and LA) had the same number of positive charges but exhibited completely different antimicrobial and hemolytic activities, which might be due to the influence of hydrophobicity. Hydrophobicity affects the ability of peptides to partition into the lipid bilayer and could be directly related to potency and host cell toxicity [34]. Therefore, hydrophobicity is another key indicator that affects the biological activity of AMPs. Increasing or decreasing the hydrophobicity outside the optimal range might result in a decrease in antimicrobial activity and an increase in blood cell lysis [34–36]. Chen et al. used leucine (L) and A to change the hydrophobicity of the antimicrobial peptide V13K_L_ and found that as the hydrophobic value increased, the hemolytic activity also increased, and it showed optimal antimicrobial activity when the hydrophobic value was appropriate [36]. In this study, the “selection index” was used as the basis for the selection of target AMPs. As shown in Table 2, LG exhibited the highest selection index, so it was selected as the target peptide for the next study. Similar to the results of this study, Baghbeheshti et al. determined the MIC values of the peptides against four kinds of Gram- bacteria, include *Pseudomonas aeruginosa* (*P. aeruginosa*) ATCC 27853, *Escherichia* *coli* (*E. coli*) ATCC 25922, antibiotic resistant *P. aeruginosa* and *E. coli* strains, and found that the antimicrobial activity of S3–4 mer-GS composed of flexible linker was 25% higher than that of S3–4 mer-DP composed of rigid linker [21]. In addition, Dipti  et al. found that r-HCV-F-MEP connected by a flexible linker could achieve a higher level of protein expression in *E. coli* than r-HCV-R-MEP (with a rigid linker) [37].

**Table 2:** The MHC (µM), GM (µM) and SI values of the peptides

Peptide	MHC^a^	GM^b^	SI^c^
(FR)_2_	16	1.62	9.87
LG	64	2.00	32
LA	32	8.00	4

^a^MHC is the concentration of peptide that causes 20% hemolysis as the minimum hemolysis concentration

^b^GM is the geometric mean of the MIC value of the peptide against bacteria. When no antimicrobial activity is detected at 32 µM, use 64 µM to calculate the selectivity index

^c^Selection index = MHC/GM, the larger the value, the higher the cell selectivity

### Integration of the target gene with the *P. pastoris* genome and the expression of 6 × His-SUMO-LG

The research results of Zhu and colleagues showed that the expression product of *P. pastoris* had better rapid performance and immune effects than the expression product of *E. coli* [38]. To date, *P. pastoris* has been successfully used to produce AMPs (such as TH2-3), enzyme preparations (such as xylanase) and vaccines (such as PpSP15) [39–41]. Therefore, this study chose *P. pastoris* as the production factory for LG.

**Fig. 2:**
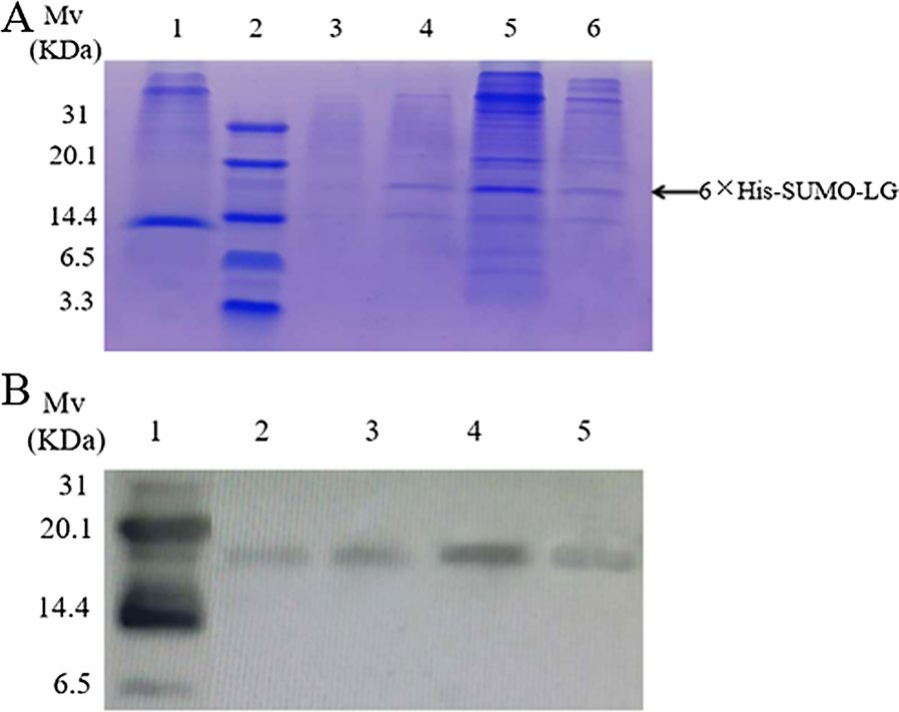
Identification of the expression of the fusion protein in *Pichia pastoris* X33. **A** Tricine-SDS-PAGE to detect the expression of fusion proteins in *P. pastoris* X33*.* Lane 1: The protein expression of empty vector pPICZαA in *P. pastoris* X33; Lane 2: Low range prestained protein marker; Lanes 3–6: The protein expression of pPICZαA-6 × His-SUMO-LG in *P. pastoris* X33. **B** Western blotting to detect the expression of fusion protein in *P. pastoris* X33*.* Lane 1: Low range prestained protein marker; Lanes 2–5: The protein expression of 6 × His-SUMO-LG in *P. pastoris* X33

In order to avoid the degradation of LG by cellular protease and to simplify the separation and purification process, tag partners were considered for use in the process of LG recombinant expression. The 6 × His tag can specifically bind to the corresponding anti-His tag antibody, and the 6 × His tag can be subjected to affinity chromatography with Ni–NTA, which makes the identification and purification process of the recombinant protein very convenient. In addition, the SUMO tag has the advantages of promoting the correct folding and soluble expression of the protein [42]. In this study, *P. pastoris* expression vector pPICZαA-6 × His-SUMO-LG was constructed, which contained the codon-optimized 6 × His-SUMO tag and LG gene (Additional file 1: Figure S1A). The results in Additional file 1: Figure S1B showed that the pPICZαA-6 × His-SUMO-LG gene was successfully integrated with the genome of *P. pastoris* X-33. The obtained positive clones were fermented, and the fermentation supernatant was analyzed by Tricine-SDS-PAGE and Western blotting. As shown in Fig. 2A and Additional file 2: Figure S2, compared with the fermentation supernatant of the empty plasmid pPICZaA, the fermentation supernatant of 6 × His-SUMO-LG had a specific band between 14.4 and 20.1 kDa. In the Western blotting assay, the specific protein could bind to the anti-His tag antibody (Fig. 2B and Additional file 3: Figure S3). In addition, the size of the band was basically the same as the theoretical size (16.22 kDa). Based on the above results, it was preliminarily determined that the target protein 6 × His-SUMO-LG had been successfully expressed.

### Effects of time, methanol concentration and culture medium pH on the yield of total protein

**Fig. 3:**
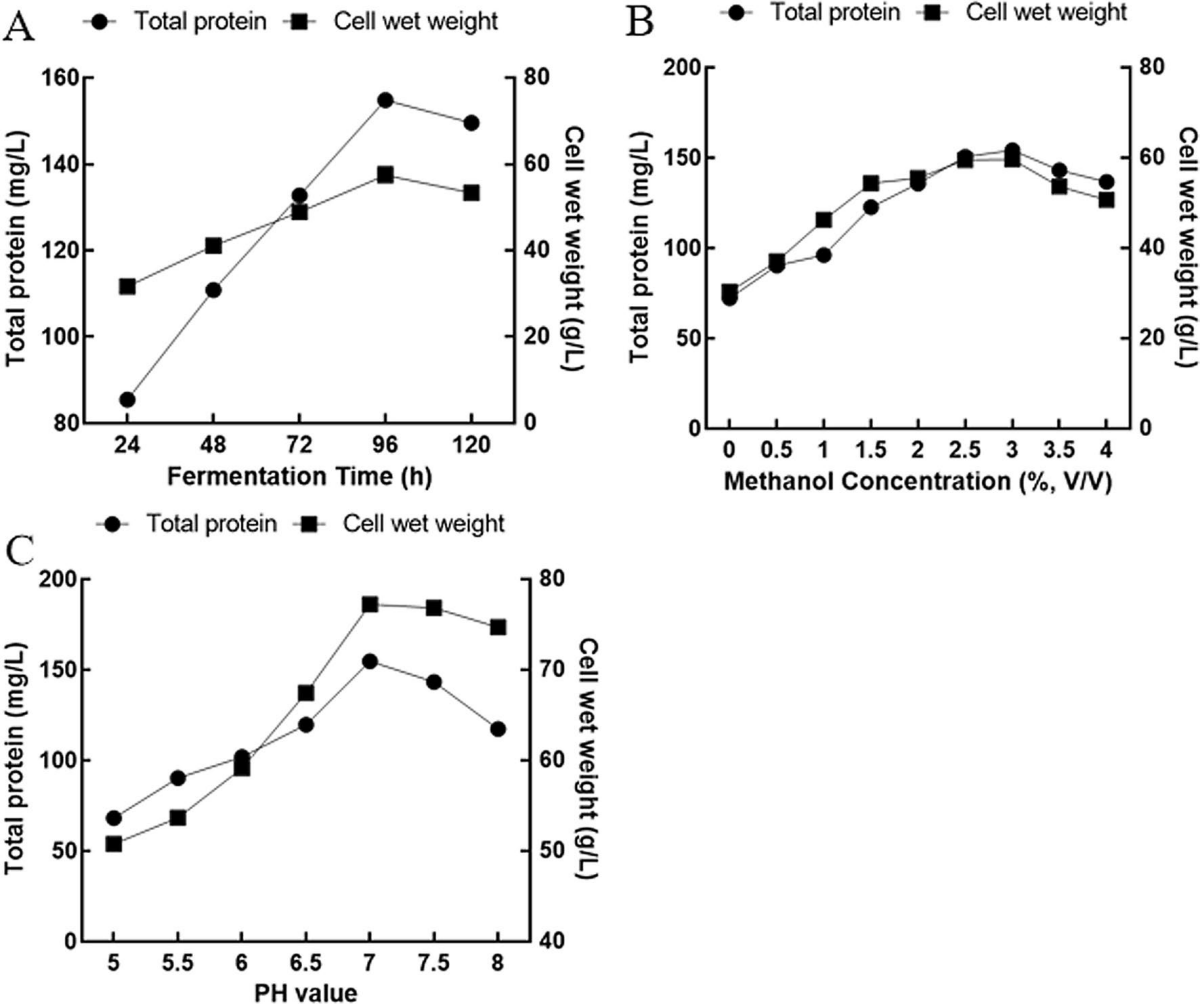
The factors affecting protein expression levels. **A** Effects of time on the yield of total protein and cell wet weight of *P. pastoris.* **B** Effects of methanol concentrations on the yield of total protein and cell wet weight of *P. pastoris.* **C** Effects of culture medium pH on the yield of total protein and cell wet weight of *P. pastoris*

As shown in Fig. 3A, the total protein expression level gradually increased with the extension of the fermentation time and reached a peak (154.8 mg/L) at 96 h after induction of fermentation, and the wet weight of *P. pastoris* continued to increase until 96 h. This might be explained as follows: with the consumption of nutrients, although the *P. pastoris* microbial cells were still growing, they were already in an aging state, and their ability to secrete proteins had decreased [43]. In addition, there were reports that the complex metabolites of *P. pastoris* might secrete enzymes that could partially degrade heterologous proteins [44]. The reasons described above led to the selection of 96 h after induction as the best induction time point for this study.

Methanol is the main carbon source and gene expression inducer of *P. pastoris*, and its concentration is directly related to the protein expression level [45]. Studies have shown that the methanol concentration required to produce recombinant protein is at least 0.5% [46]. When exploring the effect of different methanol concentrations on the protein expression level, the result found that as the methanol concentration increased, the protein expression level and the wet weight of *P. pastoris* increased until the methanol concentration reached a maximum of 3% and then began to decrease (Fig. 3B). The fermentation broth without methanol induction showed the lowest expression level and the lowest cell weight, which also illustrated the importance of methanol addition. A high concentration of methanol cause the accumulation of formaldehyde and hydrogen peroxide, which have toxic effect on *P. pastoris* microbial cells, thereby reducing their expression [47]. Therefore, it is necessary to determine the methanol level during the fermentation process to promote cell growth and increase expression while avoiding methanol toxicity.

The initial pH of the medium may affect the growth state of the host cell and thus affect the expression level of total protein. Compared with other pH values, when the initial pH of the medium was 7.0, the total protein expression and the wet weight of the host cells reached the highest level (Fig. 3C). Therefore, when the initial pH of the medium was 7.0, 96 h after induction with 3% methanol was the optimal condition for protein expression.

### Purification of 6 × His-SUMO-LG fusion protein

**Fig. 4:**
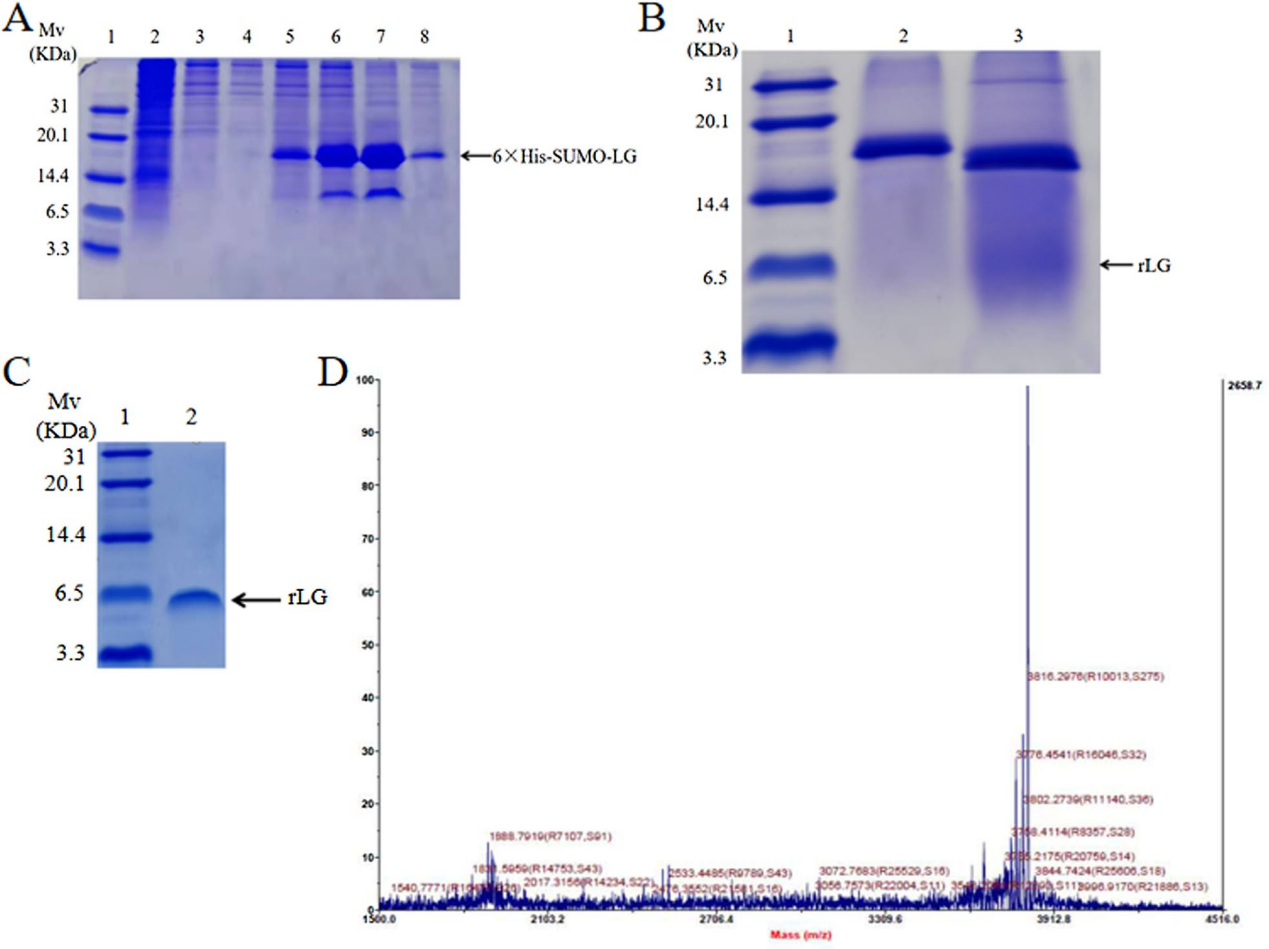
Purification of 6 × His-SUMO-LG fusion protein and rLG. **A** The 6 × His-SUMO-LG fusion protein purified by affinity chromatography and detected by Tricine-SDS-PAGE. Lane 1: Low range prestained protein marker; Lane 2: fermentation supernatant; Lane 3: penetrable apex; Lanes 4–8: The fermentation supernatant was eluted in 10 mM, 50 mM, 80 mM, 150 mM, and 250 mM imidazole. **B** Tricine-SDS-PAGE analysis of the 6 × His-SUMO-LG protein cleaved by SUMO protease. Lane 1: Low range prestained protein marker; Lane 2: 6 × His-SUMO-LG without cleavage; Lane 3: 6 × His-SUMO-LG cleaved by SUMO protease. **C** Tricine-SDS-PAGE analysis of the rLG. Lane 1: Low range prestained protein marker; Lane 2: rLG. **D** MALDI-TOF mass spectrum of purified rLG

The fermentation supernatant at 96 h after induction with 3% methanol was collected and combined with a Ni–NTA resin column to purify the 6 × His-SUMO-LG fusion protein. Figure 4A and Additional file 4: Figure S4 showed that the 6 × His-SUMO-LG fusion protein was almost completely bound to the Ni–NTA resin column. When the elution buffer concentration of imidazole was 50 mM, elation of the 6 × His-SUMO-LG fusion protein began. When the elution buffer concentration of imidazole was 80 mM and 150 mM, the maximum yield of 6 × His-SUMO-LG was reached, and the fusion protein with the highest purity was obtained when the elution buffer reached 250 mM imidazole. To facilitate subsequent research, 250 mM imidazole was selected as the optimal elution concentration, but this undoubtedly caused the loss of recombinant protein. After elution with 250 mM imidazole, the yield of 6 × His-SUMO-LG was approximately 28.63 mg/L.

### Cleavage of 6 × His-SUMO-LG and purification of recombinant LG (rLG)

In the Tricine-SDS-PAGE assay, the collected recombinant protein 6 × His-SUMO-LG was successfully cleaved by SUMO protease, and rLG was displayed at approximately 6 kDa (Fig. 4B, Fig. 4C, Additional file 5: Figure S5 and Additional file 6: Figure S6). It has been reported that AMPs form oligomeric structures at high concentrations due to rich hydrophobic amino acids and positive charges. Therefore, the phenomenon that the apparent molecular weight was 2–4 times the theoretical molecular weight was previously demonstrated in SDS-PAGE analysis [48]. This might be the reason for the difference between the apparent molecular weight of rLG and the theoretical molecular weight (3817.69 Da). MALDI-TOF analysis was used to further determine the actual size of rLG, and the results showed that the molecular weight of rLG was 3816.29 Da, which was consistent with the theoretical value of 3817.69 Da (Fig. 4D). The yield of the purified rLG peptide was 4.32 mg/L with a purity of 85.01% (Additional file 7: Figure S7). Thus, a method for producing and purifying rLG was successfully obtained in *P. pastoris*.

In this experiment, two-step Ni–NTA affinity chromatography was used to complete the purification of the fusion protein and the separation of the recombinant protein after digestion. This purification method often obtains recombinant protein with higher purity, so it is widely used in various expression systems. When Zhang et al. used the *Bacillus subtilis* expression system to produce the antimicrobial peptide T9W, the purity of T9W was > 93% after two-step Ni–NTA affinity chromatography purification [49]. In this study, after the 6 × His-SUMO-LG fusion protein was cleaved by SUMO protease and purified by nickel column, the recovery rate of rLG was approximately 64%.

### Antimicrobial and hemolytic activity of rLG

**Fig. 5:**
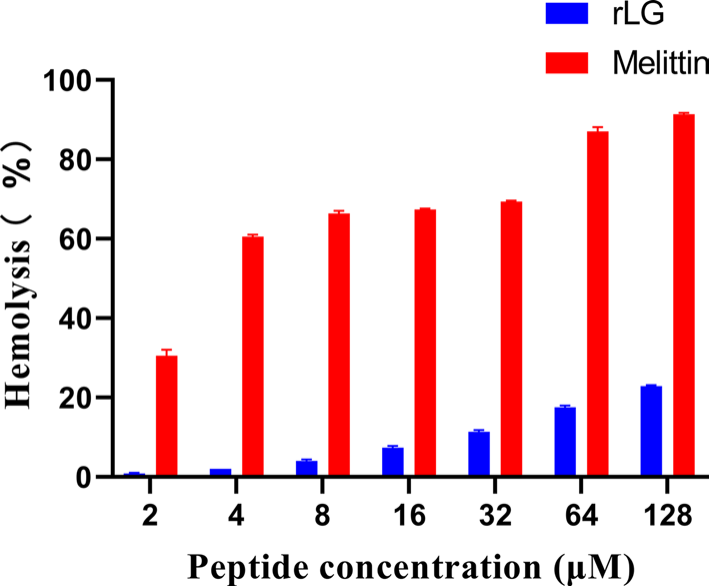
Hemolytic activity assay of rLG against human red blood cells

Another outstanding advantage of SUMO tag is that its corresponding SUMO protease can specifically recognize the tertiary structure of SUMO and cut the target protein from the fusion protein at the bisglycine terminal of SUMO, thus ensuring that there is no amino acid residue at the N-terminal of the target protein [50]. However, most protease recognize an amino acid sequence to remove a fusion tag. For example, when TEV protease removes its corresponding affinity tag, it causes S or G residues to remain at the end of the target protein, which may affect the biological activity of the target protein [51]. In this study, purified rLG showed strong antimicrobial activity against both Gram- bacteria and Gram + bacteria, and there was no significant difference in antimicrobial activity compared with chemically synthesized LG (Table 3). In addition, at the highest peptide concentration (128 μM), rLG could cause only approximately 23% of blood cells to break, which was significantly lower than melittin (91%) (Fig. 5).

**Table 3:** The MIC (μM) values of recombinant and synthetic LG

	MIC		*P*-value
	Recombinant LG	Synthetic LG	
Gram-bacteria			
*E. coli* ATCC 25922	1.77 ± 0.19	1.66 ± 0.25	0.62
*E. coli* ATCC 078	1.22 ± 0.19	1.00 ± 0	0.15
*E. coli* UB1005	1.22 ± 0.19	1.33 ± 0.25	0.62
*S. Typhimurium* C 7731	1.33 ± 0.25	1.22 ± 0.19	0.62
*S. Typhimurium* ATCC 14028	3.78 ± 0.44	3.56 ± 0.78	0.55
*P. aeruginosa* ATCC 27853	4.44 ± 1.78	4.44 ± 1.78	1
Gram + bacteria			
*S. aureus* 29213	2.00 ± 0.00	1.89 ± 0.11	0.33
*S. faecalis* 29212	1.89 ± 0.11	1.78 ± 0.19	0.55
*S. epidermidis* ATCC 12228	1.89 ± 0.11	1.89 ± 0.11	1
*S. aureus* ATCC 25923	4.44 ± 1.78	4.44 ± 1.78	1

The data were derived from three independent experiments and presented as mean ± SD. *P* < 0.05 indicated that the MIC data of the two groups was significantly different

### The influence of salt ion on the antimicrobial activity of rLG

In order to evaluate the effect of physiological concentration of salt ions on the antimicrobial activity of AMPs, this study determined the MIC of dimeric peptide rLG and monomeric peptide FR in different salt ion solutions. As shown in Table 4, Na^+^, Mg^2+^, and Ca^2+^ ions had greater impact on the antimicrobial activity of monomeric peptide FR, and Na^+^ and Ca^2+^ even caused FR to lose antimicrobial activity. The dimeric rLG in the presence of GGGGS could still maintain good activity in Na^+^ and Mg^2+^ ion solutions. The presence of salt ions caused the MIC value of monomeric peptide FR to increase by 2.44 times, while the MIC value of dimeric rLG only increased by 1.81 times. It showed that the dimeric rLG obtained by the linker GGGGS had better salt ion stability than the monomeric peptide FR.

**Table 4:** The MIC values (µM) of the peptides against *E. coil* ATCC 25922 in the presence of physiological salts

Peptide	Control^a^	MIC under physiological salt concentration^b^								
		NaCl	KCl	NH_4_Cl	MgCl_2_	CaCl_2_	ZnCl_2_	FeCl_3_	GM	Fold
FR	8	> 32	8	8	32	> 32	8	16	19.50	2.44
rLG	2	2	2	2	4	> 32	2	2	3.62	1.81

^a^The control MIC values were determined in the absence of physiological salts and serum

^b^The final concentrations of NaCl, KCl, NH_4_Cl, MgCl_2_, CaCl_2_, ZnCl_2_, and FeCl_3_ were 150 mM, 4.5 mM, 6 µM, 1 mM, 2 mM, 8 µM, and 4 µM, respectively

### The action mechanism of rLG

Most traditional antibiotics exert antimicrobial effects by inhibiting the synthesis of bacterial cell walls or DNA, so bacteria are likely to develop resistance through mutation [25]. However, AMPs usually exhibit a unique membrane destruction mechanism, and it is difficult for bacteria to develop resistance to them [52]. The positively charged amino acids of AMPs help them bind to the negatively charged bacterial membrane, penetrate the cell wall and gather in parallel on the surface of the plasma membrane. As AMPs accumulate on the plasma membrane, they form channels, leading to the permeabilization and destruction of the plasma membrane, the leakage of bacterial contents, and ultimately the death of the microbial cells [53]. Similar results were obtained in this study.

**Fig. 6:**
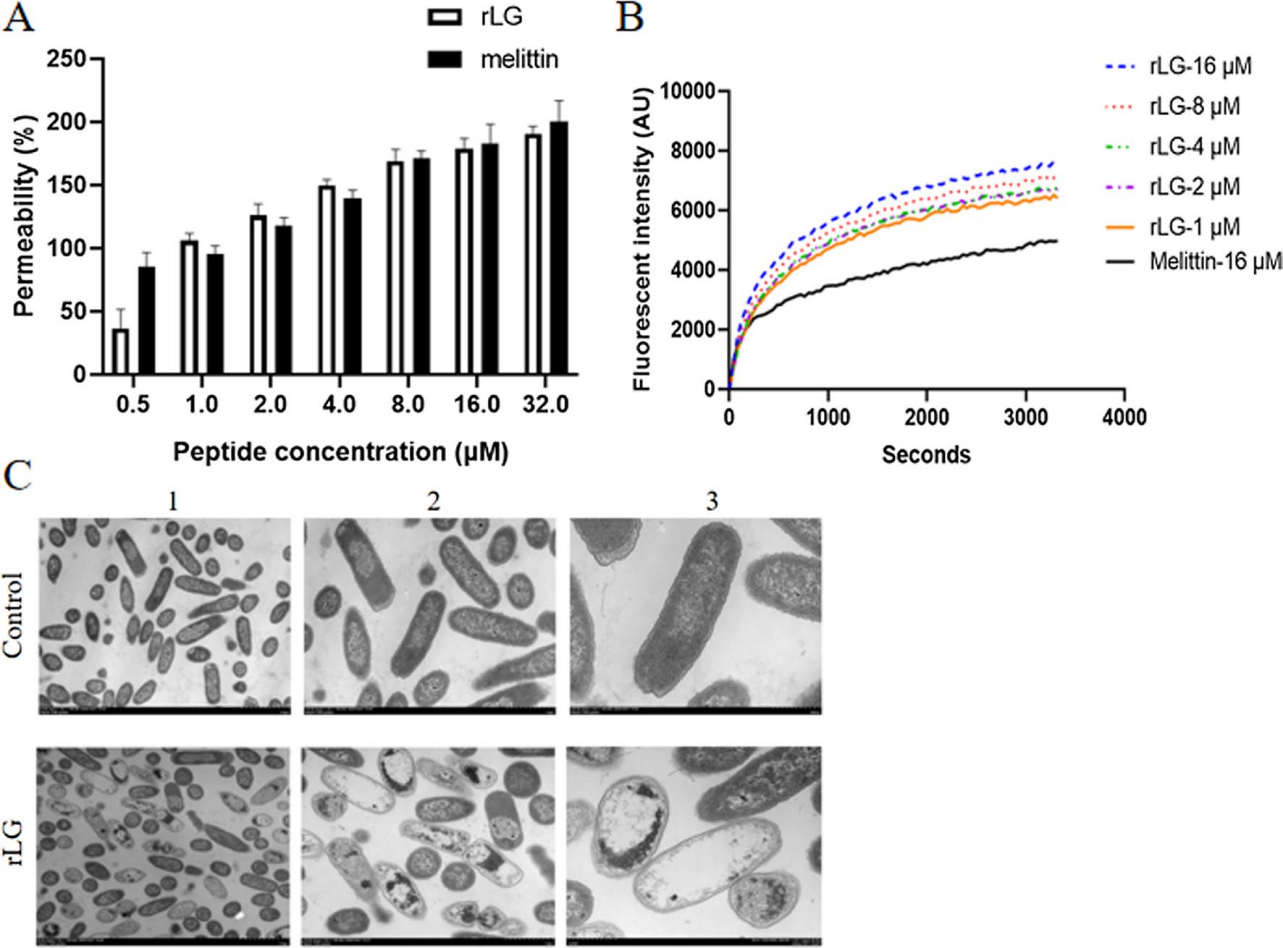
The action mechanism of rLG. **A** Outer membrane permeability of *E. coli* ATCC 25922 treated with peptides. **B** Cytoplasmic membrane potential variation of *E. coli* ATCC 25922 treated with peptides. **C** TEM images of *E. coli* ATCC 25922 treated with rLG. Lane 1: field of view magnified 5 times; Lane 2: field of view magnified 10 times; Lane 3: field of view magnified 20 times

The small molecule hydrophobic fluorescent dye 1-*N*-phenylnaphthylamine (NPN) can emit strong fluorescence in a hydrophobic environment but does not emit fluorescence in a water environment. When the bacterial cell wall is destroyed or ruptured, NPN may contact the hydrophobic environment of the cell wall, resulting in an increase in fluorescence intensity, so it can be used to indirectly evaluate the ability of rLG to destroy the bacterial cell wall. Figure 6A showed that rLG was more likely to penetrate into the bacterial cell wall of *E. coli* ATCC 25922 as the concentration increased. At low concentrations (1, 2, and 4 µM), the ability of rLG to damage or destroy the bacterial cell wall of *E. coli* ATCC 25922 was even better than that of melittin.

Recombiant LG (rLG) interacts with the plasma membrane of *E. coli* ATCC 25922 after passing through the bacterial cell wall. A cationic dye, 3, 3'-Dipropylthiadicarbocyanine iodide (DiSC_3_-5) is a dye that can penetrate into the cell membrane and exists in the cell as a nonfluorescent polymer. When the plasma membrane of the cell is destroyed, DiSC_3_-5 is released into the external environment as a monomeric and causes the fluorescence intensity to rise. Therefore, this study used fluorescence values to indicate the effect of rLG on the plasma membrane potential. It could be seen from Fig. 6B that even at the lowest tested concentration (1 µM), rLG still caused a rapid increase in fluorescence intensity, indicating that rLG could cause damage to the plasma membrane potential of *E. coli* ATCC 25922 cells, and the damage was dose- and time-dependent. This destructive effect was even stronger than that of 16 µM melittin.

To further visually show the influence of rLG on the internal morphology of *E. coli* ATCC 25922, TEM analysis was carried out. Figure 6C showed that the untreated *E. coli* membrane had a complete structure and was full of contents. After being treated with rLG, the membrane of *E. coli* was destroyed, and the cell contents flowed out.

Overall, rLG could penetrate the cell wall barrier, cause the plasma membrane to depolarize, and form holes or ion channels on the surface of the plasma membrane, leading to leakage of intracellular contents.

## Conclusions

This study partially replaced the LL37 active center amino acid and obtained a new type of AMP FR with better antimicrobial activity. To modify the expression of FR, the dimeric LG with the highest selection index was selected from among dimeric. LG significantly improved the antimicrobial activity of the monomeric peptide FR and was successfully expressed in *P. pastoris*. rLG displayed a strong antimicrobial effect by destroying the cell membrane of bacteria but had low hemolytic activity. In addition, compared with monomeric peptide FR, the ability of rLG to tolerate salt ions had been improved. This research provides new ideas for the production of modified AMPs in microbial systems and their application in industrial production.

## Materials and methods

### Strains, plasmids and reagents

The strains used for antimicrobial activity determination, *E. coli* ATCC 25922, *E. coli* ATCC 078, *E. coli* UB 1005, *Salmonella Typhimurium* (*S. Typhimurium*) C 7731, *S. Typhimurium* ATCC 14028, *P. aeruginosa* ATCC 27853, *Staphylococcus aureus* (*S. aureus*) ATCC 29213, *S. aureus* ATCC 25923, *Staphylococcus epidermidis* (*S. epidermidis*) ATCC 12228, and *Streptococcus faecalis* (*S. faecalis*) ATCC 29212, were all preserved by the Institute of Animal Nutrition, Northeast Agricultural University. The vector pPICZaA was purchased from Liuhe Huada Gene Technology Co., Ltd. (Beijing, China). Restriction endonucleases were obtained from Thermo Fisher Co., Ltd. (Waltham, USA), and SUMO protease was purchased from Gene Copoeia (Guangzhou, China). A plasmid extraction kit was obtained from Genstar (Beijing, China). The Ni–NTA Sefinose (TM) resin kit was purchased from Sangon Biotech Co., Ltd. (Shanghai, China). The purity of other chemical reagents used was of analytical grade.

### Characterization of peptides

The amino acid sequences of the peptide and their main physical and chemical parameters were shown in Table 5. In this study, R was used to replace  D in the original active center (FD) sequence of LL37 to obtain the antimicrobial peptide FR. FR was directly connected to FR to obtain (FR)_2_, and the widely used flexible linker GGGGS and rigid linker AEAAAKA were selected to connect to FR to obtain LG and LA, respectively. The antimicrobial peptides FD, FR, (FR)_2_, LG, and LA were synthesized by solid-phase synthesis by Sangon Biotech (Shanghai, China). The purity of all peptides was above 95%.

**Table 5:** Amino acid sequences and the key physicochemical parameters

Peptide	Sequences	Theoretical MW (Da)	Measured MW (Da)^a^	Net charge^b^	H^b^
FD	FKRIVQRIKDFLR	1719.11	1719.25	+ 4	0.315
FR	FKRIVQRIKRFLR	1759.19	1759.00	+ 6	0.297
(FR)_2_	FKRIVQRIKRFLRFKRIVQRIKRFLR	3502.38	3503.10	+ 12	0.297
LG	FKRIVQRIKRFLRGGGGSFKRIVQRIKRFLR	3817.69	3817.93	+ 12	0.248
LA	FKRIVQRIKRFLRAEAAAKAFKRIVQRIKRFLR	4115.06	4115.60	+ 12	0.232

^a^Molecular weight (MW) was measured by mass spectroscopy (MS)

^b^The net charge and the values of the mean hydrophobicity (H) values were calculated from http://heliquest.ipmc.cnrs.fr/cgi-bin/ComputParams.py

### Antimicrobial activity assay of peptides

The minimum inhibitory concentration (MIC) is a vital indicator for evaluating the antimicrobial activity of AMPs. The determination method refers to the improved method of Dong et al. [54]. The strains to be tested were inoculated in Mueller Hinton broth (MHB) medium and cultured at 200 rpm and 37 °C until the logarithmic growth phase, and then the number of colonies was adjusted to approximately 10^5^ CFU/mL. The 95 µL of bovine serum albumin (BSA) diluent (0.01% acetic acid and 0.2% BSA) and 5 µL of peptide (1.28 mM) were added to the first row of the 96-well plate, and 50 µL of BSA diluent was added to the other columns. After mixing in the first row, 50 µL of the mixture was aspirated and added to the second column and so on to the 11th row. After being cultured at 37 °C for 18–24 h, the minimum peptide concentration required to inhibit bacterial growth was the MIC value. All experiments were conducted at least three different times.

### Hemolytic activity assay of peptides

The safety of peptides is usually assessed by hemolytic activity. The collected blood of type B from healthy donor was centrifuged at 1000×*g* for 5 min to collect red blood cells. The red blood cells were then washed and resuspended in 10 mM PBS (pH 7.4). Fifty microliters of diluted red blood cells and an equal volume of peptide solution (2–128 µM) were incubated in a 96-well plate at 37 °C for 1 h. The red blood cell suspension treated with 0.1% Triton X-100 was used as a positive control (100% hemolysis), and untreated blood cell suspension was used as a negative control. The 96-well plate was centrifuged at 1000×*g* for 5 min at 4 °C, and then the supernatant was transferred to a new 96-well plate. The absorbance at OD_570_ was measured with a microplate reader.

### Construction of recombinant plasmid

The amino acid sequence FKRIVQRIKRFLRGGGGSFKRIVQRIKRFLR of the antimicrobial peptide LG, combined with the codon preference of *P. pastoris*, was genetically encoded. The *Eco*RI restriction site and coding gene 6 × His-SUMO were added at its 5' end, and a stop codon TAATAG and *Kpn*I restriction sites were added at the 3' end. The above process of gene synthesis and subcloning into the expression vector was completed by Beijing Liuhe Huada Gene Technology Co., Ltd.

### Transformation into *P. pastoris* cells and positive transformant selection

The constructed recombinant plasmid and empty pPICZαA plasmid were digested by *Sac*I to form a linear structure. The two plasmids were integrated with the genome of *P. pastoris* X33 by electrotransformation (2500 V, 200 Ω and 25 µF). The transformants were evenly coated on YPD solid medium containing 100 µg/mL Zeocin and cultured in a 30 °C incubator for 3–5 days. PCR experiments were carried out on all single colonies with the purpose of screening positive clones.

### Expression of the 6 × His-SUMO-LG fusion protein at the shaking flask level

The positive clones were inoculated in buffered complex glycerol medium (BMGY) and cultured in a shaker at 250 rpm and 30 °C until the OD_600_ of the culture was 2–6. The fermentation broth was centrifuged at 13,000×*g* for 10 min. Then, the collected precipitate was resuspended in buffered methanol complex medium (BMMY) medium and its concentration was adjusted to OD_600_ = 1.0.

The initial pH of the fermentation medium was controlled to 7.0, and the fermentation broth at 24, 48, 72, 96, and 120 h after induction with 3.0% methanol was sampled to explore the effect of different time points on protein expression. The methanol concentration was controlled to 0%, 0.5%, 1.0%, 1.5%, 2.0%, 2.5%, 3.0%, 3.5% and 4.0% (v/v), and the 96 h fermentation broth was collected to screen out the optimal methanol induction concentration. In addition, under induction with 3% methanol, fermentation was carried out at different initial pH values of the medium (5.0, 5.5, 6.0, 6.5, 7.0, 7.5, and 8.0). The fermentation broth was also collected 96 h after induction. The cells obtained by centrifuging 10 mL of fermentation broth at 1000×*g* for 30 min were weighed and recorded as cell wet weight. The total protein content in the fermentation supernatant was determined by Bradford assay.

### Purification of 6 × His-SUMO-LG fusion protein and release of LG

The fermentation broth was centrifuged at 95,000×*g* for 10 min at 4 °C, and then solid ammonium sulfate was gradually added to the supernatant to 70% saturation and left overnight. The precipitate was dissolved in binding buffer (containing 10 mM imidazole, pH 8.0) and dialyzed in binding buffer overnight. The concentrated dialyzed protein solution was added to the Ni–NTA column, and the fusion protein was washed with elution buffer (containing 250 mM imidazole, pH 8.0) with final concentrations of 10 mM, 50 mM, 80 mM, 150 mM, and 250 mM imidazole. The collected eluates were tested by Tricine-SDS-PAGE to screen out the optimal elution concentration.

The purified 6 × His-SUMO-LG fusion protein was mixed with SUMO protease and digestion buffer (500 mM Tris–HCl, 1.5 M NaCl, 2% NP-40, and 10 mM DTT at pH 8.0) and incubated at 4 °C for 16 h. The mixed system was passed through the nickel column again. The effluent was collected and dialyzed with a 1 kDa MWCO dialysis tube and then lyophilized. Finally, Tricine-SDS-PAGE was used to verify the purified rLG. The purity of rLG was determined by reversed-phase high-performance liquid chromatography (RP-HPLC). The molecular weight of the peptide was determined by matrix-assisted laser desorption/ionization-time of flight mass spectrometry (MALDI-TOF MS).

### The influence of salt ion on the antimicrobial activity of rLG

The salt ions were added to the BSA solution and configured into different final concentrations of salt ions (150 mM NaCl, 4.5 mM KCl, 6 µM NH_4_Cl, 8 µM ZnCl_2_, 1 mM MgCl_2_, 2 mM CaCl_2_, and 4 µM FeCl_3_) solutions. The method discribed as above was referred to to determine the MIC value of rLG against *E. coli* ATCC 25922 in the presence of different concentrations of salt ions.

### Cell wall permeabilization

The cell wall permeability was determined by monitoring the fluorescence release of the fluorescent dye NPN (Sigma, USA). *E. coli* ATCC 25922 (OD_600_ = 0.2) and NPN (10 µM) were incubated in 5 mM HEPES buffer (containing 5 mM glucose, pH 7.4) for 30 min. Then, 100 µL of peptides (0.5–32 µM) and an equal volume of bacteria were added to a 96-well plate. An F-4500 fluorescence spectrophotometer (Hitachi, Japan) was used to detect the fluorescence intensity of different wells at a 350 nm excitation wavelength and 420 nm emission wavelength. The peptide-free and polymyxin B-containing fluorescence values were set as negative and positive controls.

### Cytoplasmic membrane depolarization

DiSC_3_-5 was used to measure the change in plasma membrane potential after peptide treatment. *E. coli* ATCC 25922 (OD_600_ = 0.05) was incubated with 0.4 µM DiSC_3_-5 and 100 mM K^+^ in 5 mM HEPES buffer (containing 20 mM glucose, pH 7.4) until the fluorescence decreased stably. Subsequently, 2 mL of cell suspension was added to a sterile 24-well plate and mixed with various concentrations of peptides (2–32 µM). A fluorescence spectrophotometer (Infinite 200 pro, Tecan, China) was used to record the fluorescence values at an excitation light wavelength of 622 nm and emission light wavelength of 670 nm.

### Transmission electron microscopy (TEM) characterization

*E. coli* ATCC 25922 was cultured to the logarithmic growth phase and centrifuged to obtain the bacteria. The bacteria were resuspended twice in 0.01 M PBS (pH 7.4) and diluted to OD_600_ = 0.3. The rLG and bacteria were mixed to a final concentration of 1/2 × MIC (1 μM), and the bacteria without peptide were used as a control. Both samples were incubated at 37 °C for 1 h. The incubated sample was centrifuged at 2500×*g* for 5 min and washed three times with 0.01 M PBS. Two milliliters of 2.5% glutaraldehyde buffer was added to the bacterial pellet, and the suspension was gently blown and fixed at 4 °C overnight. The cells were washed with 0.01 M PBS and fixed in 1% osmate buffer for 1 h. Then, different concentrations of ethanol were used for gradient dehydration (50%, 70%, 85%, 95% and 100%). Finally, the sample was prepared into ultrathin sections through the process of soaking and embedding, and a Hitachi H-7650 TEM (Hitachi, Japan) was used for observation.

### Statistical analyses

The mean and standard deviation (SD) were calculated using SPSS 16.0. Data were expressed as the mean ± SD. The comparison between each group of data was performed by ANOVA. *P* < 0.05 was defined as a significant difference. All experiments were conducted at least three times.

## Fundings

This project was supported by the Natural Science Foundation of China (32030101 and 31872368), the Natural Science Foundation of Heilongjiang Province (TD2019C001) and the China Agriculture Research System of MOF and MARA.

## Supplementary Information

**Additional file 1: Figure S1.** Construction of the pPICZαA-6 × His-SUMO-LG expression vector. (A) Schematic diagram of the pPICZαA-6 × His-SUMO-LG expression vector. (B) Identification of the expression vectors pPICZαA and pPICZαA-6 × His-SUMO-LG by PCR amplification. Lane 1: Low range prestained protein marker; Lane 2: Expression vector pPICZαA clone; Lanes 3–7: Expression vector pPICZαA-6 × His-SUMO-LG clones.

**Additional file 2: Figure S2.** Tricine-SDS-PAGE to detect the expression of fusion proteins in *P. pastoris* X33 (original figure of Fig. 2A)*.*

**Additional file 3: Figure S3.** Western blotting to detect the expression of fusion protein in *P. pastoris* X33 (original figure of Fig. 2B)*.*

**Additional file 4: Figure S4.** The 6 × His-SUMO-LG fusion protein purified by affinity chromatography and detected by Tricine-SDS-PAGE (original figure of Fig. 4A).

**Additional file 5: Figure S5.** Tricine-SDS-PAGE analysis of the 6 × His-SUMO-LG protein cleaved by SUMO protease (original figure of Fig. 4B).

**Additional file 6: Figure S6.** Tricine-SDS-PAGE analysis of the rLG (original figure of Fig. 4C).

**Additional file 7: Figure S7.** Determination of the purity of rLG by reversed-phase high-performance liquid chromatography.

## Data Availability

All datasets generated and analyzed during this study are included in this published article and its additional files.
