# Supplementary figures and images for: Design and heterologous expression of a novel dimeric LL37 variant in Pichia pastoris
Source: Microb Cell Fact. 2021 Jul 23;20:143. doi: 10.1186/s12934-021-01635-x (PMC8305873; doi:10.1186/s12934-021-01635-x)

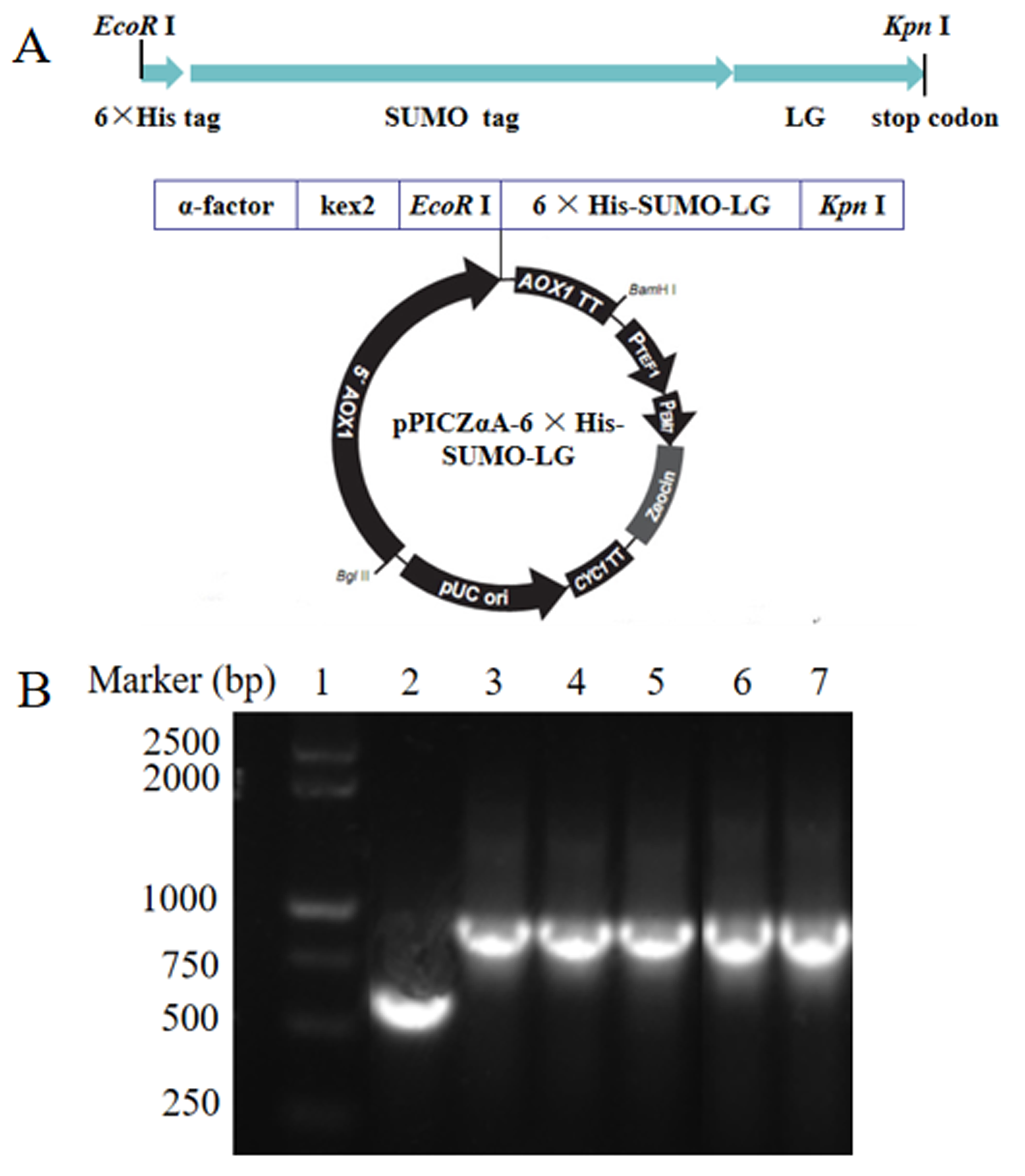

Supplement: Supplementary file 1 — Additional file 1: Figure S1. Construction of the pPICZαA-6 × His-SUMO-LG expression vector. (A) Schematic diagram of the pPICZαA-6 × His-SUMO-LG expression vector. (B) Identification of the expression vectors pPICZαA and pPICZαA-6 × His-SUMO-LG by PCR amplification. Lane 1: Low range prestained protein marker; Lane 2: Expression vector pPICZαA clone; Lanes 3–7: Expression vector pPICZαA-6 × His-SUMO-LG clones. [file 12934_2021_1635_MOESM1_ESM.tif]

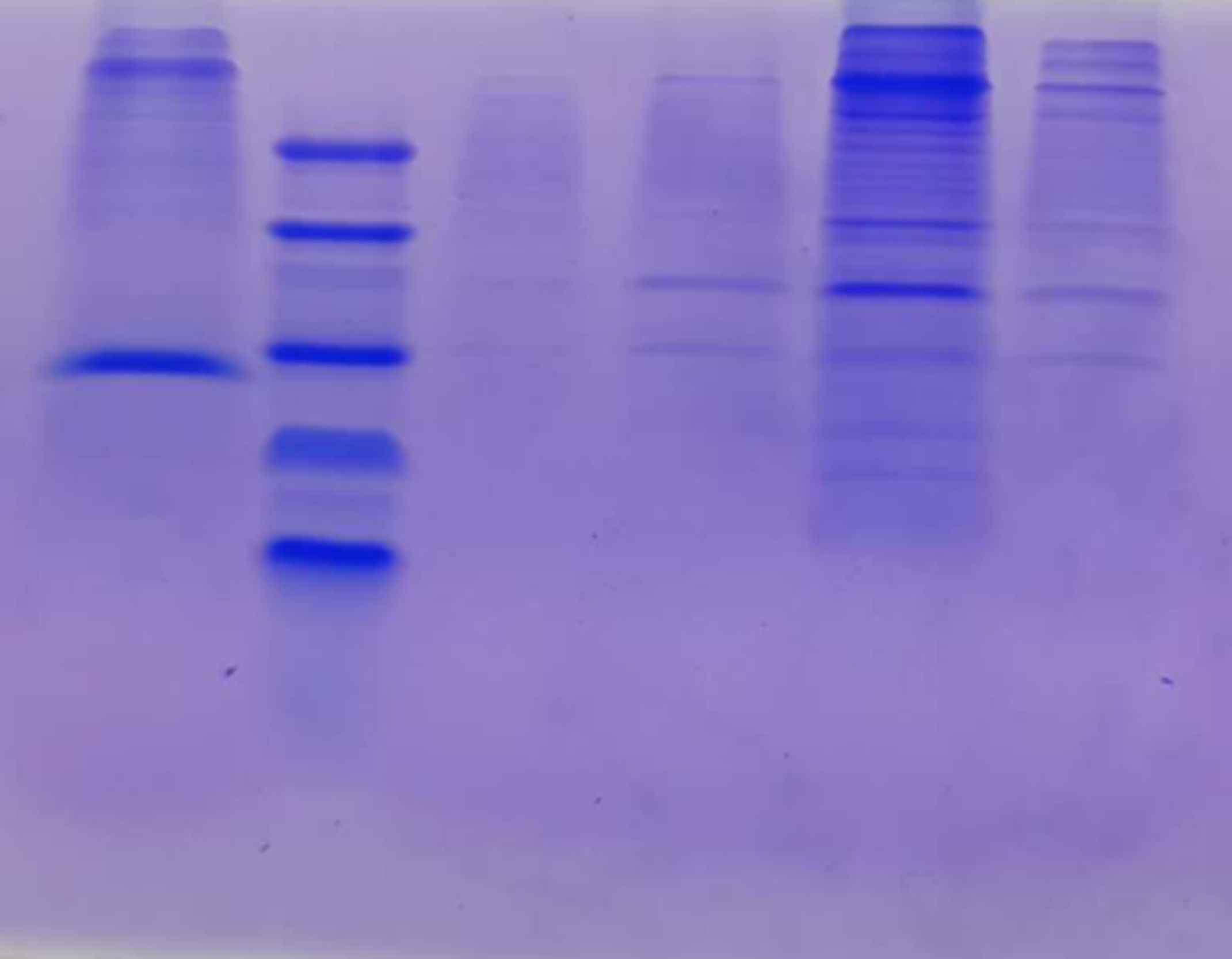

Supplement: Supplementary file 2 — Additional file 2: Figure S2. Tricine-SDS-PAGE to detect the expression of fusion proteins in P. pastoris X33 (original figure of Fig. 2A). [file 12934_2021_1635_MOESM2_ESM.tif]

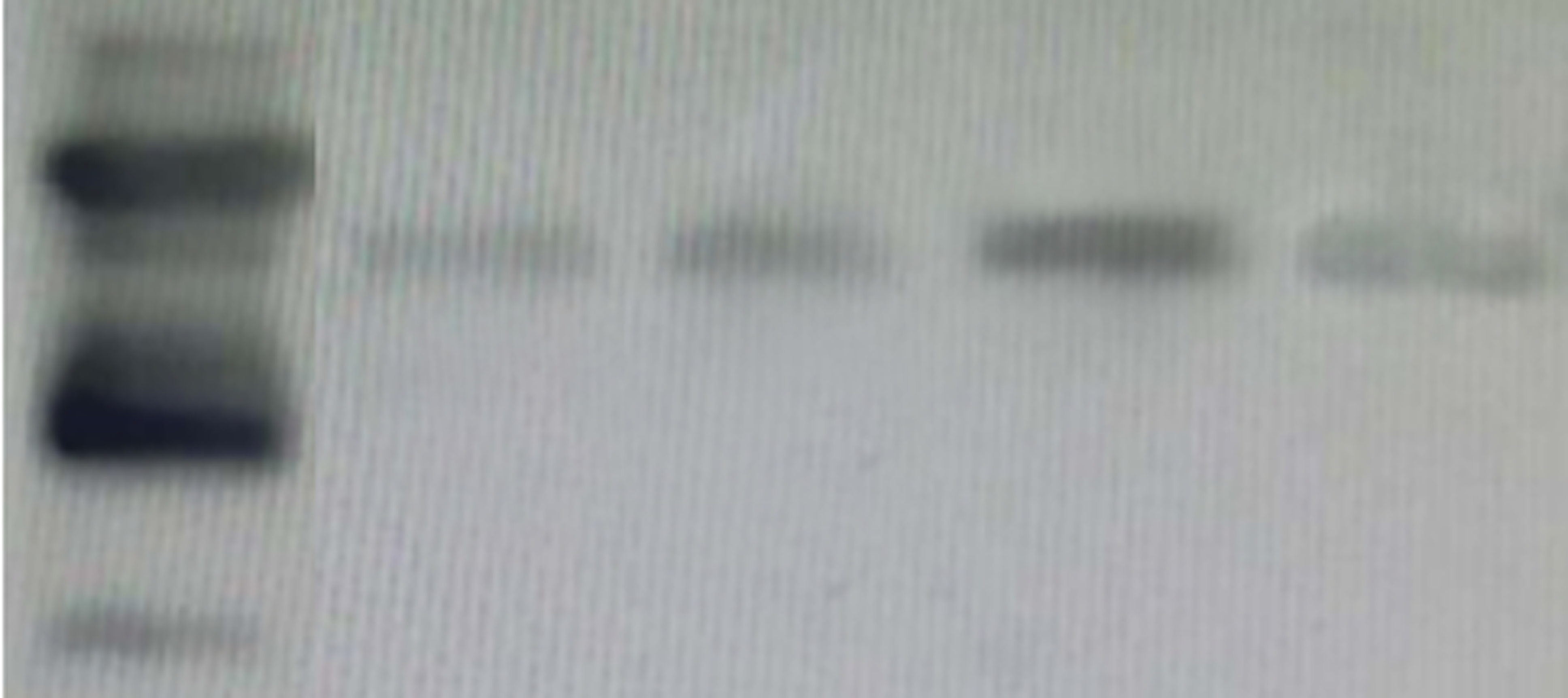

Supplement: Supplementary file 3 — Additional file 3: Figure S3. Western blotting to detect the expression of fusion protein in P. pastoris X33 (original figure of Fig. 2B). [file 12934_2021_1635_MOESM3_ESM.tif]

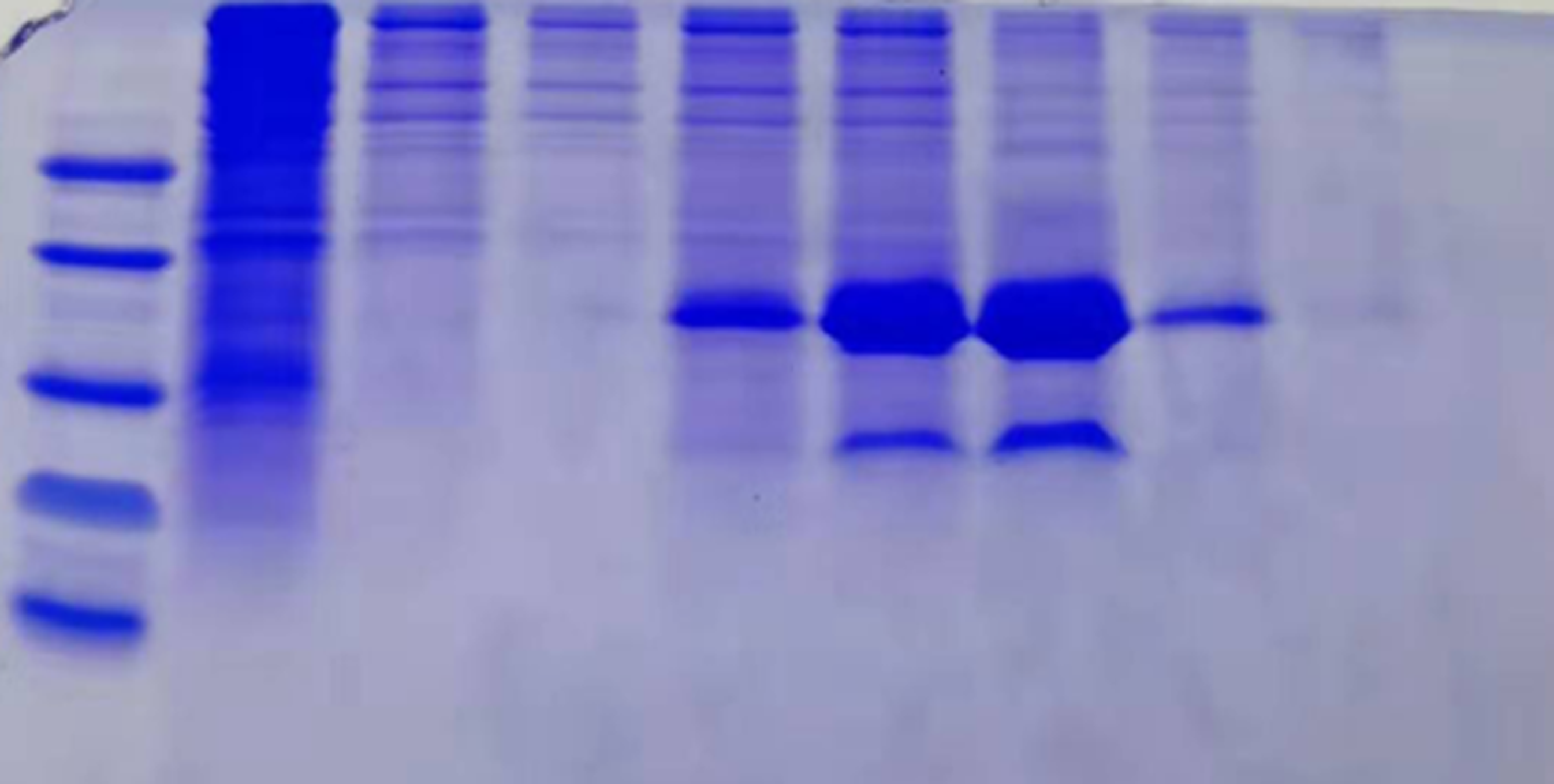

Supplement: Supplementary file 4 — Additional file 4: Figure S4. The 6 × His-SUMO-LG fusion protein purified by affinity chromatography and detected by Tricine-SDS-PAGE (original figure of Fig. 4A). [file 12934_2021_1635_MOESM4_ESM.tif]

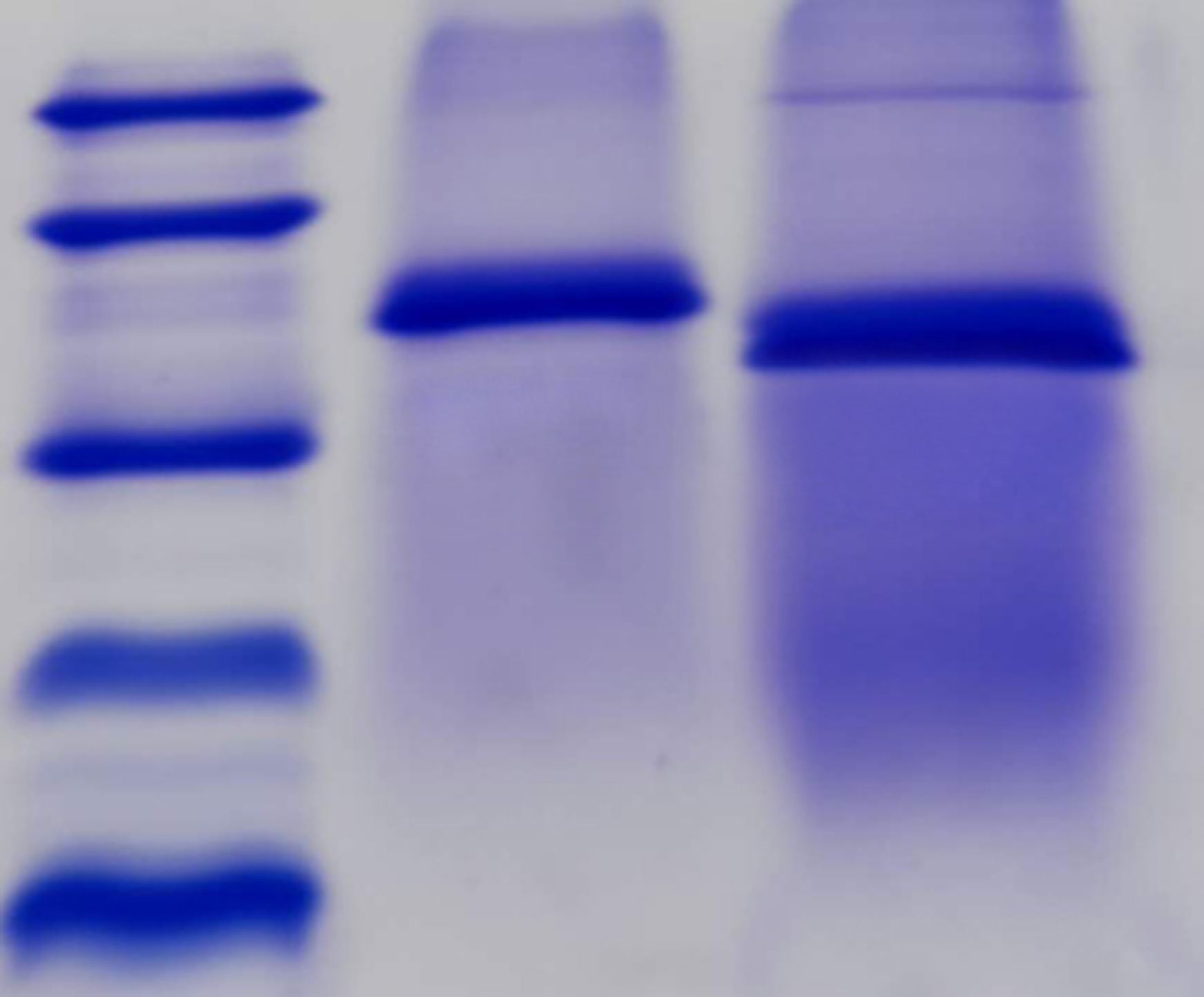

Supplement: Supplementary file 5 — Additional file 5: Figure S5. Tricine-SDS-PAGE analysis of the 6 × His-SUMO-LG protein cleaved by SUMO protease (original figure of Fig. 4B). [file 12934_2021_1635_MOESM5_ESM.tif]

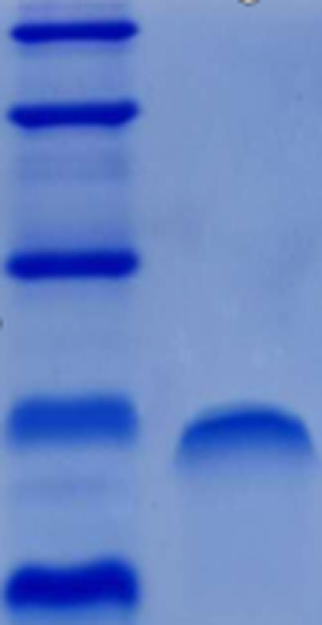

Supplement: Supplementary file 6 — Additional file 6: Figure S6. Tricine-SDS-PAGE analysis of the rLG (original figure of Fig. 4C). [file 12934_2021_1635_MOESM6_ESM.tif]

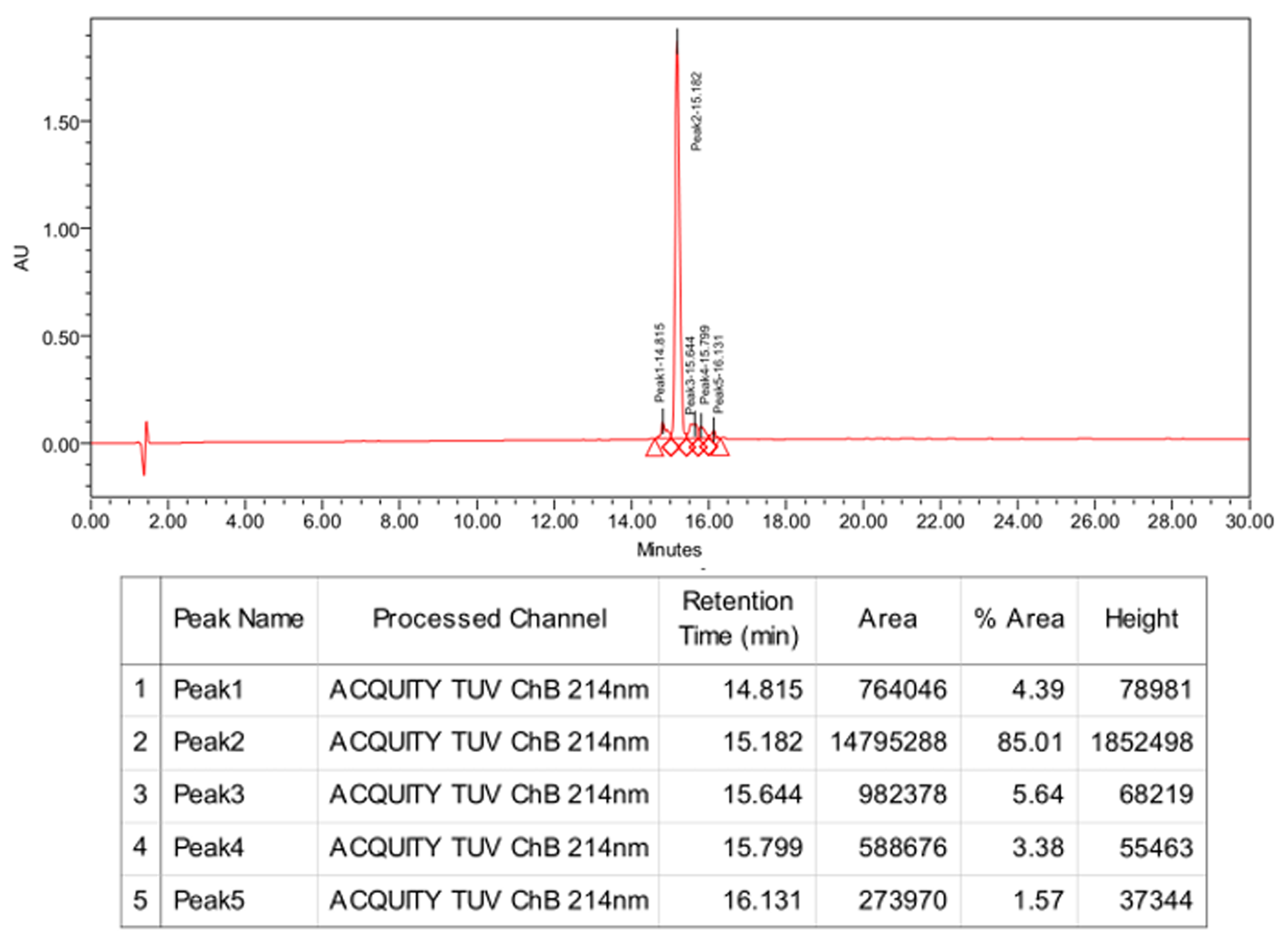

Supplement: Supplementary file 7 — Additional file 7: Figure S7. Determination of the purity of rLG by reversed-phase high-performance liquid chromatography. [file 12934_2021_1635_MOESM7_ESM.tif]
